# Supplementary figures and images for: Detection of SARS-CoV-2-Specific Secretory IgA and Neutralizing Antibodies in the Nasal Secretions of Exposed Seronegative Individuals
Source: Viruses. 2024 May 27;16(6):852. doi: 10.3390/v16060852 (PMC11209246; doi:10.3390/v16060852)

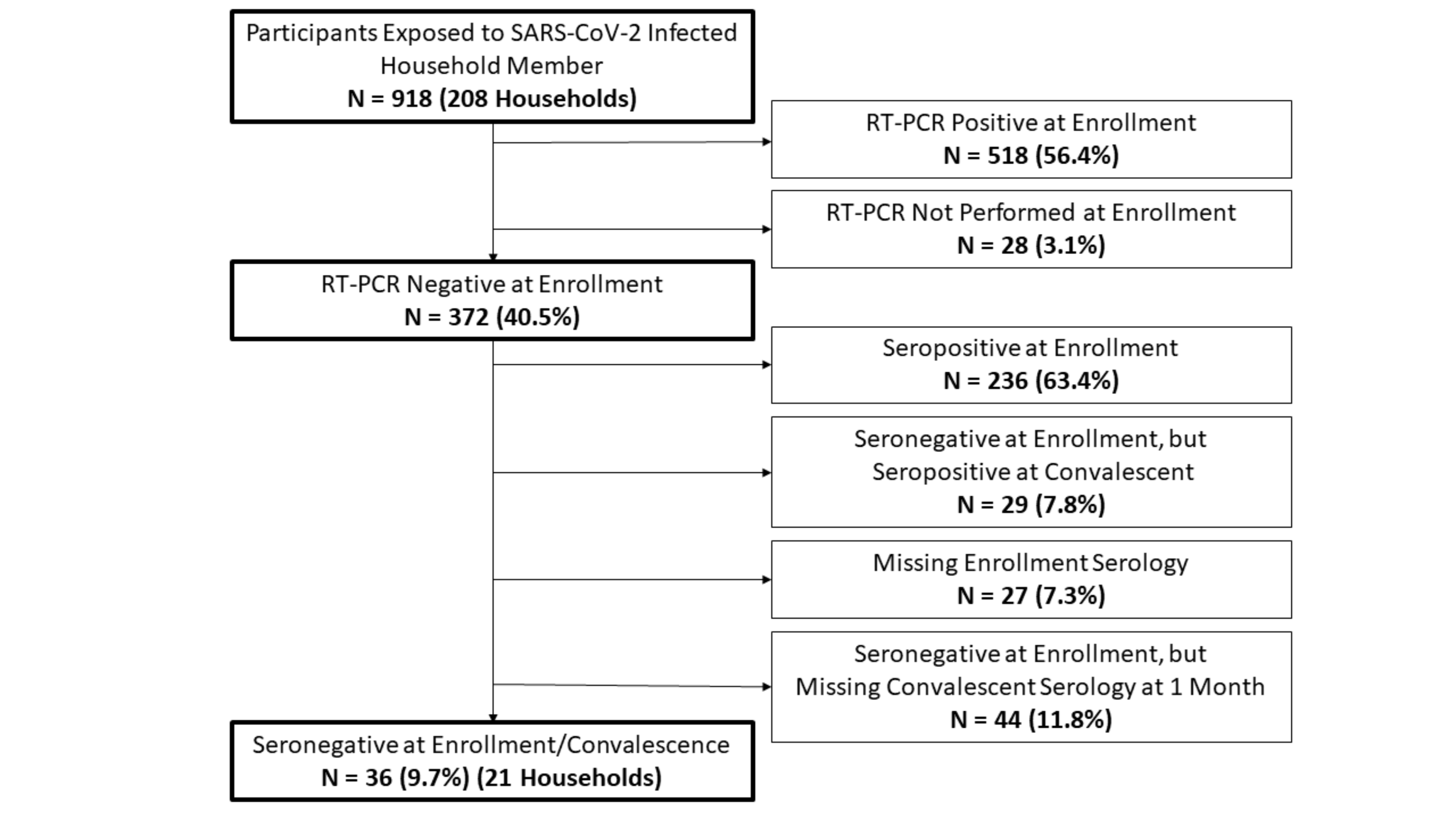

Supplement: Supplementary file 1 [file viruses-16-00852-s001.zip › Fig S1.tiff]

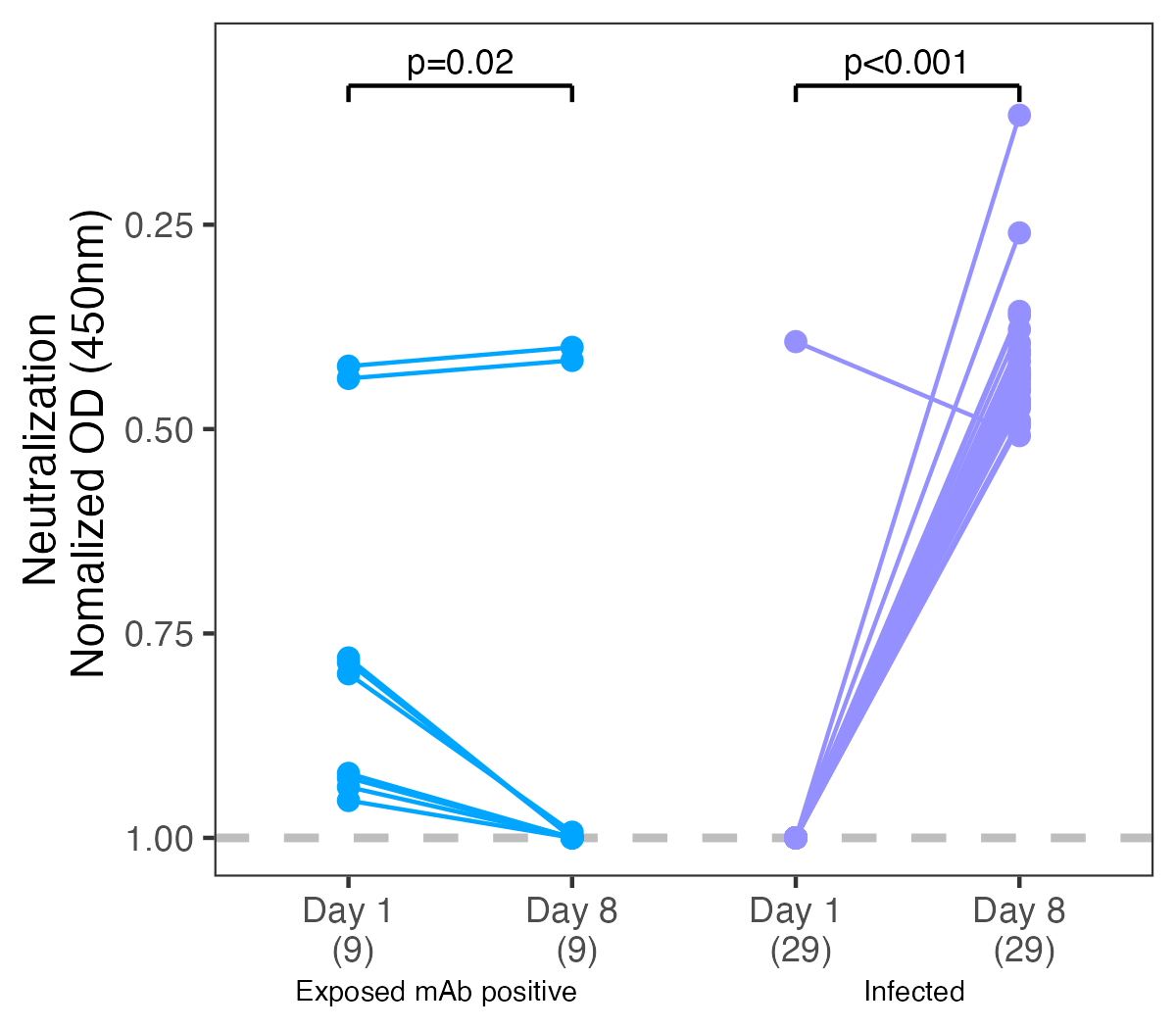

Supplement: Supplementary file 1 [file viruses-16-00852-s001.zip › Fig S2.tiff]
